# Enablers and barriers of COVID‐19 vaccination efforts in Nepal

**DOI:** 10.1002/puh2.67

**Published:** 2023-02-09

**Authors:** Sunit Chhetri, Srista Manandar, Sunny Chhetri, Astha Acharya, Swapnil Regmi, Dil Bahadur Gurudhami, Thinley Dorji

**Affiliations:** ^1^ Department of Internal Medicine B.P. Koirala Institute of Health Sciences Dharan Nepal; ^2^ Department of Internal Medicine Central Regional Referral Hospital Gelegphu Bhutan

**Keywords:** COVID‐19, health‐care financing, immunization, Nepal, pandemic, SARS‐CoV‐2, vaccine development

## Abstract

Nepal, a landlocked country with a population of 29.1 million faced great socioeconomic, financial, and social hardships during the Coronavirus disease 2019 (COVID‐19) pandemic. Nepal had increasing cases of COVID‐19 despite multiple lockdowns and travel restrictions. Nepal reported 1,000,775 cases and 12,019 deaths till early November 2022. Timely government decisions, strategic political diplomacy, flexible vaccine policy, and multiple loan deals helped Nepal secure the COVID‐19 vaccine from India in a commendable time frame. With the existing structure of the primary health care settings and experience of having implemented childhood vaccination campaigns, Nepal rolled out the COVID‐19 vaccines across the nation on 27 January 2021. However, the delta wave was a devastating blow to South Asia with a high number of hospitalizations and deaths and a complete disruption of the COVID‐19 vaccine supply from India. The government ran a relentless effort in COVID‐19 vaccination and provided full vaccination to 33% of the total population by the end of 2021 and 81.1% by May 2022. The booster doses were administered beginning in 2022, but the campaign efforts have been lethargic with a coverage of only 29.2% and a lack of keen support from the public. The emergence of new variants of the virus continues to pose a challenge in controlling the disease and reopening the country to pre‐pandemic levels of socioeconomic activities.

## INTRODUCTION

After Coronavirus disease 2019 (COVID‐19) was declared a pandemic in March 2020, great efforts were made worldwide to contain the spread of the disease with strict movement restrictions, social distancing, and nationwide lockdowns but could not avoid significant morbidity and mortality waves in many countries. To mitigate the health, social, and economic impacts of COVID‐19, vaccines were developed in record time by December 2020 and early 2021 [1].

As of June 2022, 11 vaccines were granted Emergency Use Listing by the World Health Organization (WHO) and 38 vaccines have been approved for use in different countries [2]. Around the globe, 12,885,748,541 COVID‐19 vaccines have been administered by early November 2022 [3]. In Nepal with a population of 29.1 million [4], eight different types of vaccines have been approved by the government but only six have been deployed for vaccination till now [2]. A total of 59,431,338 doses have been administered by November 2022 covering complete doses of 81.06% of total the population [3].

In Nepal, the first case of COVID‐19 was officially announced on 23 January 2020 [5], and by November 2022, Nepal had reported 1,000,775 cases and 12,019 deaths [3] To fight back against the rapid spread of COVID‐19, Nepal enforced two nationwide and multiple regional lockdowns in many states [6, 7]. Widespread closure of all the private and public offices along with movement restrictions and social distancing helped Nepal control the spread of COVID‐19 to some extent, but there was a widespread loss of jobs and livelihood. Despite people facing hardships, Nepal had exemplary efforts in launching the COVID‐19 vaccination becoming one of the first nations to go ahead with population vaccination. The start of the vaccination campaign was commendable, but the follow‐up to the campaign lacked energy and enthusiasm [7]. This article summarizes the efforts in rolling out COVID‐19 vaccines in Nepal.

## NEPAL'S HEALTH AND VACCINATION SYSTEM

Nepal, a country in the lap of the Himalayas, after promulgating the new constitution in 2015 was restructured to a federal republic with seven provinces. The health system has also been redesigned providing increased autonomy and responsibility to the local governments [8]. Besides, being a signatory of the Alma‐Ata declaration of 1978 and the recent Astana Declaration, Nepal has continuously reformed its primary health care (PHC) system [9]. In doing so, Nepal has expanded its health infrastructure to include 125 hospitals, 198 PHC centers, 3808 health posts, and other public facilities by 2017/18 [10]. But still, accessibility to PHC settings is dismal with only 61.8% of the population having access to health services within 30 min of travel with an alarming gap between urban (85.9%) and rural (59%) population [11].

Within the framework of PHC, Nepal launched the Expanded Program on Immunization in 1979, now known as National Immunization Programme. Initially, launched in 3 districts with 2 antigens bacille Calmette‐Guérin (BCG) and diphtheria, pertussis, and tetanus (DPT), the program was rapidly expanded to include all 75 districts. By 1988, the immunization schedule included oral polio vaccine and measles. Subsequently, the monovalent hepatitis B (HepB) vaccine was introduced in 2003, *Haemophilus influenzae type B* (Hib) vaccine was introduced in 2009, and the pneumococcal conjugate vaccine (PCV) and inactivated polio vaccine‐intramuscular (IPV‐IM) were introduced in 2015 [12]. Nepal was declared polio‐free in 2014 underlining one of the successes in vaccine‐preventable diseases [13]. The Nepal Demographic and Health Survey (NDHS) 2016 reported that 78% of children aged 12–23 years have received all eight basic vaccinations—one dose each of BCG and Measles‐Rubella and three doses each of DPT–HepB–Hib and polio vaccine. The coverage of the National Immunization Programme has increased to 99% in NDHS 2016 [12]. The details of vaccination coverage under the National Immunization Programme are shown in Table 1.

**TABLE 1:** Vaccination coverage of the National Immunization Schedule in Nepal, 2016 [12].

Type of vaccine		Vaccination coverage (%)
BCG		98
Polio	1	98
	2	95
	3	88
DPT–HepB–Hib	1	97
	2	94
	3	88
Pneumococcal vaccine	1	73
	2	94
	3	88
IPV‐IM		70
Measles		90

Abbreviations: BCG, bacille Calmette‐Guérin; DPT, diphtheria, pertussis, and tetanus; HepB, monovalent hepatitis B; Hib, *Haemophilus influenzae type B*; IPV‐IM, inactivated polio vaccine‐intramuscular.

Besides the infrastructure and framework of the vaccination campaign available through the PHC system, the National Health Policy of Nepal 2019 aims to provide access to basic health‐care services (BHCSs) free of charge, whereas non‐BHCSs are to be covered through the social health insurance [14]. Nepal rolled out the COVID‐19 vaccination campaign as early as January 2021.

## COVID‐19 VACCINATION IN NEPAL

### Planning for COVID‐19 vaccination

The Government of Nepal, after having imposed 5‐month long nationwide lockdown from 12 March to 21 July 2020 [6], was under great pressure to act early and procure the COVID‐19 vaccines. On 20 December 2020, the Government of Nepal amended the Drugs Act 2035 through an ordinance, permitting the registration of new drugs and vaccines at the Department of Drug Administration (DDA) for emergency use. Such an amendment opened the door for manufacturers to import new products not listed in the WHO‐approved vaccines [15]. In early 2021, the Ministry of Health and Population with technical support from the WHO Country Office (WCO) and partners formulated the National Deployment and Vaccination Plan (NDVP) with an aim to secure enough doses through the COVID‐19 Vaccines Global Access (COVAX) Facility to vaccinate at least 20% of the population at the highest risk of COVID‐19 [16]. In addition, a high‐level political delegation from Nepal visited India on 14 January 2021 to discuss the procurement of the COVID‐19 vaccine [15].

As a part of planning for the procurement of vaccines, the Government of Nepal sought multiple loans to finance them. The loan deal was signed with the World Bank amounting to US$29 million on April 2020, US$75 million on March 2021, and US$18 million on January 2022 for the COVID‐19 Emergency Response and Health Systems Preparedness (CERHSP) Project [17]. On 22 July 2021, the Asian Development Bank approved a loan of US$165 million for the Government of Nepal [18].

Nepal launched its first vaccination campaign in January 2021 with the one million doses of AstraZeneca COVISHIELD donated by India, Nepal's southern neighbor. Backed by the strong presence of PHCs at the grassroots level, the experience of nationwide mass vaccination campaigns in the past and the advertisement of first doses of vaccination by high‐profile individuals through multiple national media outlets helped in the acceptance of vaccines in the first phase of vaccination. The vaccination campaign included posters, social media messages, auto‐generated caller ringback tones in state‐owned telecommunication service provider, Nepal Telecom, and advocacy through health personnel and community health volunteers.

### Vaccine types and doses procured

The Government of Nepal approved eight COVID‐19 vaccines: AstraZeneca COVID‐19, Sinopharm's Vero Cell (BBIBP‐CorV), Janssen (Johnson & Johnson), Pfizer vaccine, Moderna vaccine, Sinovac‐CoronaVac, Covaxin, and Sputnik V [2]. Covaxin manufactured by India and Sputnik V manufactured by Russia were approved for emergency use but were never deployed. Nepal has received vaccines from multiple donor countries and the COVAX facility as shown in Table 2. Besides, Nepal had also bought vaccines from multiple sources, but cost was not disclosed for diplomatic reasons.

**TABLE 2:** The mobilization of the Coronavirus disease 2019 (COVID‐19) vaccines in Nepal (updated till May 2022) [7, 18].

**Vaccine type**	**Under scheme**	**Doses (million)**
**AstraZeneca and COVISHIELD AstraZeneca vaccine**	Donated by countries (India, Bhutan, Japan, UK, Maldives, Switzerland, Canada, Denmark, France, and Italy)	8.1
	COVAX facility	6.387
	Bought from Serum Institute of India (SII) at $4 per dose	2.0
**Sinopharm BIBP vaccine**	Donated by China	3.8
	Bought from China at undisclosed fee	10.0
	Bought from China with COVAX cost sharing scheme	5.936
**Janssen vaccine**	Donated by USA and Germany	3.704
**Pfizer‐BioNtech vaccine**	Donated by USA	0.765
	COVAX facility (9.2 million)	1.5^a^
**Moderna vaccine**	COVAX facility	3.712
	Bought with COVAX cost sharing scheme	4.0
**Sinovac‐CoronaVac vaccine**	Donated by China	4.056
**TOTAL**		53.96

^a^COVAX facility pledged 9.2 million doses of the Pfizer‐BioNTech vaccine on February 2022 of which only 1.5 million has been dispatched till the end of May 2022.

### Vaccination process (implementation)

Nepal launched the COVID‐19 vaccination campaign on 27 January 2021 with AstraZeneca COVISHIELD donated by India. A nationwide campaign led to the completion of the first phase of vaccination on 5 March 2021 with 438,000 individuals, mainly frontline workers and health workers, receiving the vaccine [7, 18].

In February 2021, the government bought 2 million doses of the COVISHIELD vaccine from the Serum Institute of India at US$4 per dose. The first consignment of 1 million doses was delivered in February 2021. On 7 March 2021, Nepal received 348,000 doses of COVISHIELD through the COVAX facility. The second phase of vaccination started on 7 March 2021 and vaccinated 1.3 million people. Although the second phase aimed to inoculate all adults above 65 years of age, it came to an abrupt halt as the Serum Institute of India could not supply the remaining 1 million doses of vaccine as India was engulfed by a deadly surge of cases during its second wave. The second dose of COVISHIELD was completed only in August 2021 when the vaccines were donated by Bhutan and Japan [7, 18].

The vaccine pledged by China, Sinopharm BIBP Vaccine (Vero cell vaccine), arrived in Nepal on 29 March 2021, and the government resumed the inoculation campaign on 7 April 2021. The Sinopharm vaccine was used to inoculate all essential workers aged between 18 and 59 years followed by anyone willing and aged between 40 and 59 years totaling up to 289,000 people. The second dose of the Sinopharm vaccine was administered from 16 to 25 May 2021 [7, 18].

The second wave of the pandemic in April–June 2021 delayed the vaccination campaign as Nepal could not secure or procure vaccines. The vaccination campaign gathered pace only in July 2021, when the government received donations of vaccines and purchased 10 million doses of Sinopharm through a nondisclosure agreement. An additional 5.9 million doses of the Sinopharm vaccine were bought through COVAX's cost‐sharing scheme [7, 18].

On 12 July 2021, the government received 1.5 million doses of the Johnson & Johnson vaccines from the United States under the global COVAX facility. The Janssen vaccines were administered to people aged between 50 and 54 years, those with disabilities, refugees residing in Nepal, migrant workers, and students going abroad. Nepal further received 100,620 doses of the Pfizer‐BioNtech vaccine from the United States through the COVAX facility that was administered to people with immunocompromised conditions [18]. The COVAX facility provided 3.7 million doses of the Moderna vaccine in the first lot in December 2021, which were used to immunize 1.7 million children aged 12–17 years in 57 districts (of a total of 77 districts). The second lot of 640,000 doses of the Pfizer vaccine obtained through the COVAX facility was used to immunize children in the remaining 20 districts [7, 18].

With the availability of vaccines and accessing their suitability, vaccines were administered to all of the targeted population in a phased manner. With existing framework in place, Nepal mainly administered vaccines through the available tertiary hospitals, district hospitals, and PHCs. The arduous task of maintaining cold chain during vaccine transport to remote regions of Nepal was possible with support from United Nation Children's Fund (UNICEF) Nepal. Vaccines were transported by airways, roadways, and even, by porters and dispensed from the locally available health centers. Female Community Health Volunteers (FCHV) and other community volunteers from rural and remote Nepal played special roles in communicating and convincing the unreached population for vaccination [19, 20]. By the end of 2021, vaccine availability and timely second dose were not an issue as shown in Table 3 [7, 18]. However, full vaccine coverage was only 15% in September 2021 and only 33% by the year‐end. The reasons for this poor coverage were lack of knowledge about the vaccination campaign and the importance of vaccination, skepticism about the vaccines, inconveniences of time and place of vaccination, especially among daily wage earners and rural population, and overall stagnant advertising policy by the government [21]. By end of 2021, the government had decided to administer booster doses only after vaccine coverage reached 40% of the total population [7].

**TABLE 3:** Enablers and challenges to the Coronavirus disease 2019 (COVID‐19) vaccination in Nepal.

Enablers
Wide network of PHC centers, dissemination of vaccine through locally available health‐care centers
Experience from annual nationwide vaccination campaigns
Timely amendment of new drug act to authorize use of emergency products
Early action to secure vaccines from COVAX facility
Diplomacy to secure vaccine from the neighbors and donors
Financial deals from the World Bank and Asian Development Bank to purchase vaccines
Effective advertisement of vaccination campaign
Support from UNICEF Nepal to maintain cold chain for vaccine transport

Barriers
Stagnant approach of vaccine advertisement after initial efforts
Inability to overcome vaccine skepticism among recipients
Inconvenient time for vaccination especially among daily wage worker
Overall lack of enthusiastic approach for booster dose campaign
Lack of trust in seeking health care from government centers

### COVID‐19 vaccine booster doses

Nepal started administering booster shots from 17 January 2022. For primary vaccination, Nepal mostly followed the homologous vaccination technique, but for booster dose, the mix‐and‐match technique was endorsed. Initially, the government administered booster shots to those with a gap of 6 months from the second dose of COVID‐19, but by April 2022, booster shots were administered to those with a gap of more than 3 months as recommended by the National Immunization Advisory Committee [22].

The booster doses were provided in a phased manner with frontline workers receiving them first before it was allowed for the general population beginning on 12 February 2022. In the first 52 days, data from the Ministry of Health and Population showed that only 5% of the total population was inoculated. This was an early red flag for the booster dose vaccination campaign [23], and the uptake of booster doses has been only 29.2% as of early November 2022 [3].

## IMPACT OF COVID‐19 VACCINATION IN NEPAL

**FIGURE 1:**
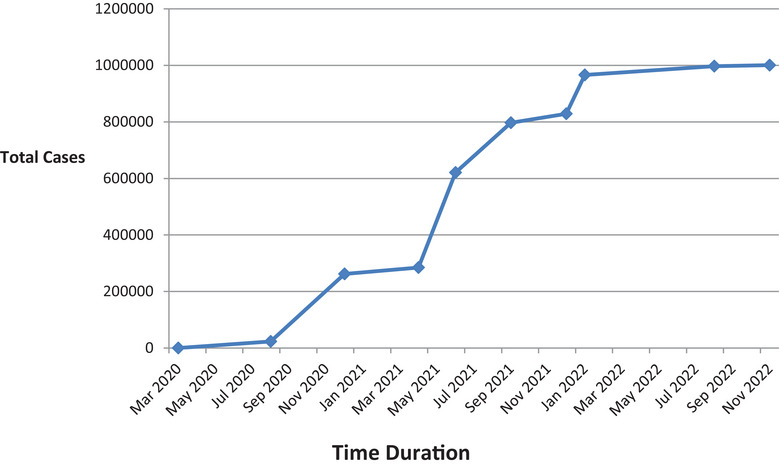
Timeline of rise in number of the Coronavirus disease 2019 (COVID‐19) cases in Nepal (updated till Nov, 2022) [3].

**FIGURE 2:**
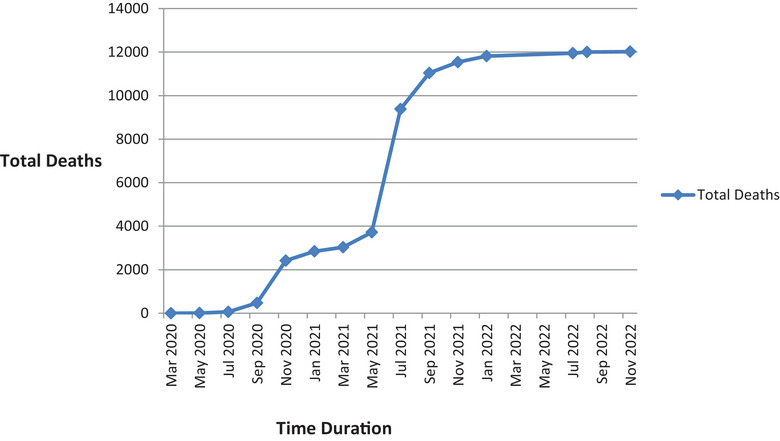
Timeline of number of the Coronavirus disease 2019 (COVID‐19) deaths in Nepal (updated till Nov, 2022) [3].

The second wave of pandemic with the delta variant in April 2021, when the vaccine coverage with a single dose was 6–7%, led to 8000 additional deaths totaling 11,000 deaths by the end of the second wave [7]. Meanwhile, the omicron variant and its sub‐lineages, though more infectious, supposedly caused the third wave in the country when the vaccine coverage was 33% and had no significant impact on morbidity and mortality as illustrated in Figures 1 and 2 [3, 24].

The education sector was also severely impacted by the COVID‐19 pandemic. Schools were closed in March 2020, and students remained out of school for 20 months. Only by November 2021, the government was confident in opening the schools after securing enough vaccines for the fluent progress campaign [25]. Similarly, schools were again closed in 11 January 2022 amid fear of the third wave of pandemic due to the Omicron variant but opened 1 month later in mid‐February 2022 with no such risk mostly due to ongoing vaccination campaign [26].

The economic sector faced similar great adversities due to multiple lockdowns across countries amid the COVID‐19 pandemic. Nepal was hit hardest economically from mid‐March to mid‐August 2020, during the first pandemic with strict lockdown, with fear of COVID‐19 among general public at its peak prior to the vaccination campaign. With improving vaccination coverage and decreasing fear among public, the economic sector made gradual recovery [27].

Nepal continues its efforts in COVID‐19 vaccination with two vaccine clinical trials being conducted in 2022 with the aim to mass produce vaccines in the country in the future. The vaccines in Phase III clinical trial are developed by Sanofi in partnership with GlaxoSmithKline (GSK) and WestVac Biopharma [28].

## CONCLUSION

Nepal utilized its PHC setting and the National Immunization Programme infrastructure for the nationwide COVID‐19 vaccination campaign. Prompt adaptive changes in national policies and regulations, strong political will, proactive vaccine diplomacy, and timely arrangement of multiple financial loans helped Nepal secure the COVID‐19 vaccines. The government made commendable efforts, and enough vaccine doses were secured to provide full vaccination and, also, booster doses. However, follow‐up on the vaccination campaign was rather stagnant and was lagging behind the desired milestones. With reopening measures and resumption of travel and transport, Nepal still faces the challenges of newer potent variants of COVID‐19.

## AUTHOR CONTRIBUTIONS

All authors were involved in the conception of the idea. Sunit Chhetri, Srista Manandhar, and Thinley Dorji drafted the initial version of manuscript. All authors played a major role in the acquisition of all relevant data. Sunit Chhetri and Thinley Dorji were involved in a critical review of the article. All authors have approved the final draft for publication.

## CONFLICT OF INTEREST STATEMENT

Thinley Dorji is a member of the editorial board of this journal. He was excluded and blinded from all processes leading to the acceptance of this article.

## Data Availability

Data derived from public domain resources. The data that support the findings of this study are available mostly on the website of the Department of Health Services, Nepal and the Kathmandu Post, Nepal. These data were derived from the resources available in the public domain as mentioned in the reference section of the manuscript.
